# Integrating Metabolomics and Network Analysis for Exploring the Mechanism Underlying the Antidepressant Activity of Paeoniflorin in Rats With CUMS-Induced Depression

**DOI:** 10.3389/fphar.2022.904190

**Published:** 2022-06-13

**Authors:** Chaofang Lei, Zhigang Chen, Lili Fan, Zhe Xue, Jianbei Chen, Xihong Wang, Zhen Huang, Yinian Men, Mingzhi Yu, Yueyun Liu, Jiaxu Chen

**Affiliations:** ^1^ School of Traditional Chinese Medicine, Beijing University of Chinese Medicine, Beijing, China; ^2^ Dongfang Hospital Affiliated to Beijing University of Chinese Medicine, Beijing, China; ^3^ Guangzhou Key Laboratory of Formula-Pattern of Traditional Chinese Medicine, School of Traditional Chinese Medicine, Jinan University, Guangzhou, China

**Keywords:** paeoniflorin, CUMS, metabolomics, SLC6A4, TNF, IL6, network analysis, Slc6a3

## Abstract

**Background:** Paeoniflorin (PF) represents the major bioactive constituent of the traditional Chinese medicine plant *Paeonia suffruticosa* (Ranunculaceae), which has a long history as a folk medicine in Asian. Paeoniflorin, a bitter pinene monoterpene glycoside, has antidepressant effects, but its potential therapeutic mechanism has not been thoroughly explored.

**Methods:** Experimental depression in rats was established by the chronic unpredictable mild stress (CUMS) combined with orphan method, and the efficacy of paeoniflorin on depression was evaluated by the sucrose preference test and open field test. The antidepressant mechanism of paeoniflorin was investigated by metabolomic and network pharmacology. The relevant pathways of biomarkers highlighted in metabolomics were explored, and the possible targets of paeoniflorin in the treatment of depression were further revealed through network analysis. The binding activity of paeoniflorin to key targets was verified by molecular docking.

**Results:** Metabolomics showed that rats with CUMS-induced depression had urine metabolic disorders, which were reversed by paeoniflorin through the regulation of metabolic pathways. Metabolites that play a key role in the function of paeoniflorin include citric acid, thiamine monophosphate, gluconolactone, 5-hydroxyindoleacetic acid and stachyose. Key predicted targets are *SLC6A4*, *TNF*, *IL6* and *SLC6A3*. An important metabolic pathway is the Citrate cycle (TCA cycle).

**Conclusion:** Network integrative analysis in this study showed that paeoniflorin could improve depressive-like symptoms in model rats with CUMS-induced depression and overall correct the disordered metabolic profile through multiple metabolic pathways.

## 1 Introduction

Depression has become a major public health issue in the world. The World Health Organization ranks depression third top cause of global disease burden, and it is expected to rank first by 2030 (Malhi and Mann 2018). Depression is a high-risk factor for stroke, dementia, hypertension and other diseases. Studies have shown that depression persists 6 months after stroke, which not only seriously affects neurological repair in stroke patients, but also delays the recovery process, thereby increasing the risk of recurrent stroke and increasing mortality (Nickel and Thomalla 2017; Dong et al., 2021). Depression also increases the risk of dementia and Alzheimer’s disease, with potentially higher effects than other known risks (e.g., smoking, high blood sugar, high cholesterol, high homocysteine, vascular factors, etc.,) (Diniz 2018; Alexopoulos 2019; Santabarbara et al., 2019; Weisenbach et al., 2019). In addition, depression is an independent risk factor for hypertension, increasing the risk of hypertension by 42% (Meng et al., 2012; Zhang et al., 2018). Therefore, there is an urgent need to explore a more effective strategy and to find potential drug targets for depression.

Traditional Chinese medicine has been applied for depression treatment for millennia, with demonstrated efficacy, good safety profile and distinct features. Paeoniflorin (PF), a constituent of *P. suffruticosa* Andr., *Paeonia lactiflora* Pall. or *Paeonia veitchii* Lynch, is a monoterpene glycoside. PF possesses multiple activities such as anti-inflammatory, hypoglycemic, antithrombotic, anticonvulsant, cardioprotective and immunomodulatory effects (Xiang et al., 2020; Zhang et al., 2020; Zhou et al., 2020). Recent pharmaceutical studies have shown that paeoniflorin exerts significant antidepressant effects, by upregulating monoaminergic neurotransmitter levels, inhibiting hypothalamic-pituitary-adrenal axis hyperfunction, promoting neuroprotection, promoting hippocampal neurogenesis, upregulating brain-derived neurotrophic factor levels, inhibiting inflammatory responses and reducing nitric oxide levels, among others (Wang et al., 2021). Zhong et al. (2018) found that paeoniflorin ameliorated chronic stress-induced depression-like behavior and neuronal damage in rats by activating the ERK-CREB pathway (Zhong et al., 2018). Chang et al. (2015) demonstrated that paeoniflorin exerts an antidepressant effect by reducing neuroinflammation by inhibiting CASP-11-dependent pyroptosis signal transduction (Chang et al., 2015). Some researchers found that *P. lactiflora* could regulate CUMS-induced metabolic disorders in rats (Tian et al., 2021). However, studies have not yet elucidated the relationship between the metabolites, metabolic pathways and targets of paeoniflorin antidepressant effects.

Network pharmacology is an emerging discipline that explores the associations of drugs, targets and diseases through network information analysis. It provides a novel research potentially combining the traditional “one target, one drug” strategy. This has been transformed into a “multi-target, multi-component” research method (Meng et al., 2020). This method is consistent with the holistic view of TCM theory. Metabolomics represents a fast-growing field of “omics” that could help comprehensively assess endogenous metabolites in biological samples, making it possible to identify true regulatory models of molecular metabolites *in vivo*.

In recent years, urinary metabolomics has become an important research method for the discovery of non-invasive biomarkers (Kwon et al., 2020). Urine is the end product of the body’s metabolic network, which is rich in metabolites and can reflect the overall metabolic level in the body. Urine has the characteristics of non-invasive and easy-to-obtain specimens, which can reflect the physiological and pathological states of the body and the response of the body to drugs and toxic substances. It is an ideal object for scientific research (Zhang et al., 2016). Currently, LC-MS is the most widely used technique for urine metabolomics analysis (Wu et al., 2022).

This study employed a combination of network pharmacology and LC-MS-based untargeted metabolomics to explore urinary biomarkers of paeoniflorin in depression therapy. Metabolic pathway and comprehensive metabolic network analyses could help identify major pathways involved in CUMS-induced depression and uncover potential therapeutic targets. This study not only provides a solid foundation and a new perspective for the characterization of paeoniflorin’s anti-depressant effects, but also provides a novel approach for studying the rationality of the compatibility of other traditional Chinese medicines.

## 2 Material and Methods

### 2.1 Animals and Treatments

Thirty-two SPF male Sprague-Dawley rats (200 ± 20 g) were provided by Beijing Vital River Laboratory Animal Technology Co., Ltd. [animal license No. SCXK (Beijing) 2016-0011]. Animal housing was carried out under a 12/12 h light-dark cycle at 22°C ± 2°C and 50%–60% humidity, with water and rodent chow *ad libitum*. Following 1 week of adaptive feeding, the animals were randomized into four groups, including the control (NC, *n* = 8), model (CUMS, *n* = 8), paeoniflorin (PF, *n* = 8) and fluoxetine (FLU, *n* = 8) groups. The control group had four rats per cage, and the other groups had one animal per cage. Assays involving animals had approval from the Institutional Animal Care and Use Committee of Beijing University of Chinese Medicine, in compliance with the Animal Welfare Guidelines (BUCM-4-2014070401-3001).

From the fourth week of model establishment, all animals were given intragastric administration 30–60 min before model establishment. The 32 rats received the following medications: 1) Control rats, no stressor and intragastric (ig) treatment with distilled water (10 ml/kg body weight); 2) CUMS group, modeling and ig treatment with distilled water (10 ml/kg body weight); 3) PF group, modeling and ig treatment with paeoniflorin (10.0 mg/kg/d, 10 ml/kg body weight) (Yajing 2021); 4) FLU group, modeling and ig treatment with fluoxetine (2.0 mg/kg/d, 10 ml/kg body weight) (Hou et al., 2020).

### 2.2 Chemicals and Materials

Paeoniflorin was produced by Chengdu Lemeitian Pharmaceutical Technology (Chengdu, China). Paeoniflorin purity: HPLC ≥ 98%. Fluoxetine hydrochloride was provided by Shanghai McLean Biochemical Technology (Shanghai, China). Sucrose was provided by Sinopharm Chemical Reagent (Shanghai, China).

LC/MS-grade methanol, acetonitrile and water were purchased from Fisher Chemical (Roskilde, Denmark). 2-Propanol were purchased from Merck (Darmstadt, Germany). Aormic acid were purchased from CNW (Shanghai, China). 2-Chloro-L-Phenylalanine were purchased from Adamas-beta (Shanghai, China).

### 2.3 Chronic Unpredictable Mild Stress Model Establishment

**FIGURE 1:**
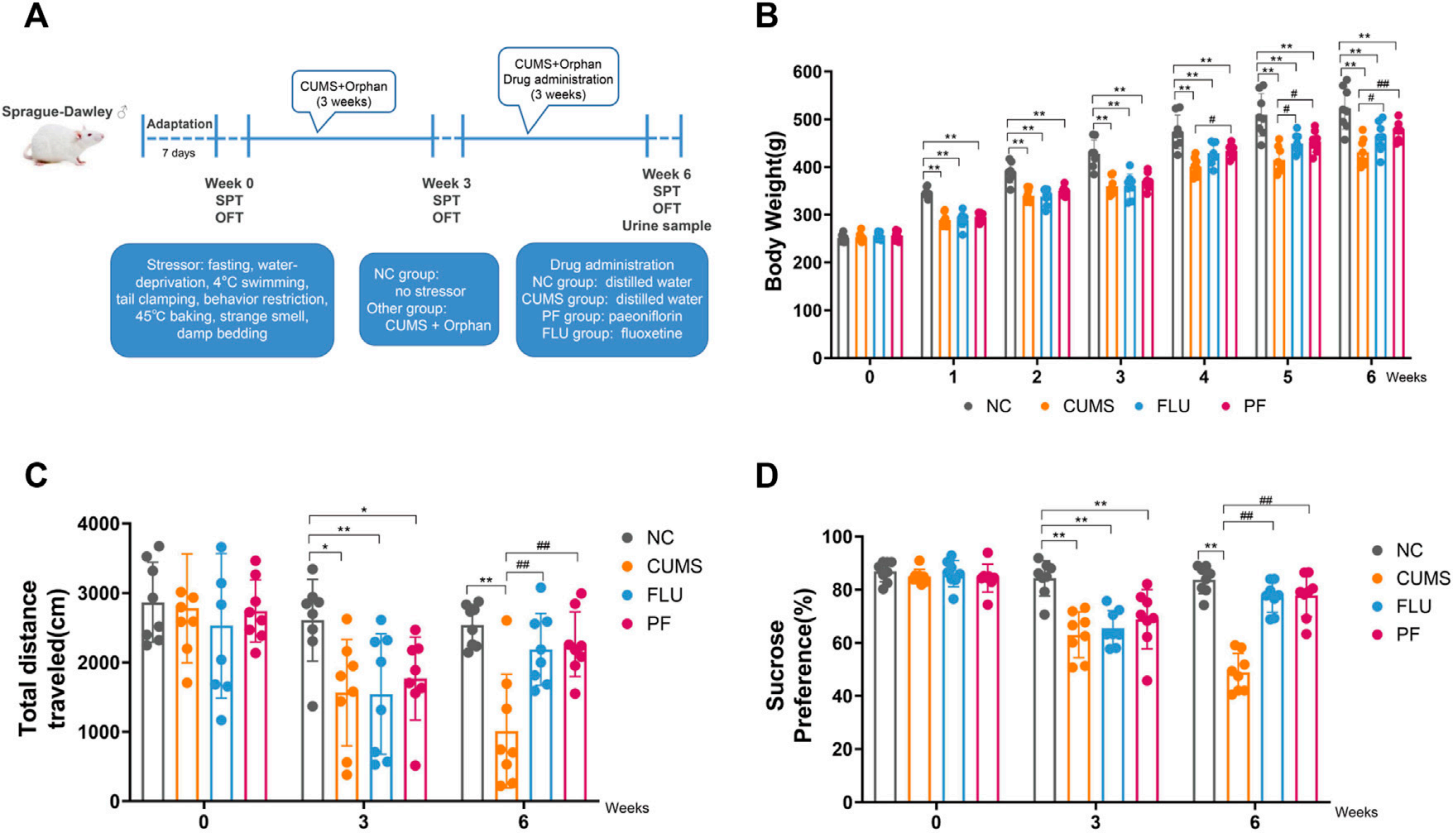
**(A)** Flow chart for establishing a CUMS-induced depression model. **(B)** Body Weights in various groups. **(C)** Total distance in various groups. **(D)** Sugar consumption rates in various groups. Data are mean ± standard deviation, *n* = 8. *p < 0.05, **p < 0.01 versus NC group; ^#^ *p* < 0.05, ^##^ *p* < 0.01 versus CUMS group.

Except for control rats, all groups underwent daily exposures in random order to one of the following chronic unpredictable mild stress for 6 weeks: 24-h fasting, 24-h water-deprivation, 5-min ice water swimming at 4°C, 1-min tail clamping (1 cm from the tail root), 3-h behavior restriction, 5-min hot baking (45°C), 24-h strange smell (glacial acetic acid sprayed on the litter for 24 h) and 24-h damp bedding. CUMS was randomly performed as summarized in Table 1. Figure 1A described the experimental schedule.

**TABLE 1:** CUMS random stress timetable.

Weekday	Fasting	Water-deprivation	4°C swimming	Tail clamping	Behavior restriction	45°C baking	Strange smell	damp bedding
Monday	√							√
Tuesday		√						
Wednesday			√					
Thursday				√				
Friday					√			
Saturday						√		
Sunday							√	

### 2.4 Body Weight Measurements and Behavioral Tests

Body weights in all rats were recorded weekly, and behavioral tests were performed at weeks 0, 3, and 6. Behavioral tests included sucrose preference test and open field test, and all animals underwent three behavioral tests. The first behavioral experiment was carried out after 1 week of adaptive feeding, and then the modeling was started. The second behavioral experiment was carried out 3 weeks after modeling. Modeling continued for 3 weeks after the second behavioral test, and medication was administered during the modeling period. Therefore, in addition to the behavioral testing time, the model was modeled for a total of 6 weeks. A third behavioral experiment was conducted 6 weeks after modeling. Rat urine samples were collected after the third behavioral experiment.

The open field test was carried out for evaluating behavioral characteristics in each group such as autonomous activity, exploration and anxiety. The open field box was opened in the room, and a camera was placed above the center of the open field box and connected to a computer for real-time video recording. Prior to the actual test, the animals were placed in the room 30 min for adaptation. For the test, the animals were placed in the center of the open field box, and their movements were recorded with a high-definition camera for 5 min. Ethovision v3.0 was utilized to assess rat behavior in various groups. After each experiment, 75% ethyl alcohol and clean water were utilized for cleaning the open field box successively. Upon smell dissipation, the next test was carried out; the entire assay was performed in a quiet environment.

The sucrose preference test was carried out in two steps. First, the animals were housed in single cages, with 2 flasks of 1% sucrose solution per cage and *ad libitum* drinking for 24 h. Next, one flask of 1% sucrose solution and one of purified water were placed per cage, for a 24-h *ad libitum* drinking. In the training period, the animals underwent a 24-h fasting for food and water. In the following step, a flask of 1% sucrose solution and one of purified water were placed per cage, with the animals allowed a 1-h *ad libitum* drinking. The sucrose solution and purified water were recorded pre- and post-experiment. Sucrose preference was derived as follows: Sucrose preference (%) = (sucrose intake/total intake) × 100. This assay is mainly utilized for assessing anhedonia level in rats.

### 2.5 LC-MS-Based Urine Metabolomics

#### 2.5.1 Urine Sample Preparation

After the last behavioral experiment, all animals underwent individual housing in metabolic cages to collect 12-h urine samples. Then, urine specimens were centrifuged (3,500 g, 4°C) for 10 min, and supernatants were kept at −80°C. Urine samples for LC-MS were prepared as follows. First, 200 μl of each urine specimen was placed into a 1.5-ml tube and mixed with 800 μl of extraction solution [methanol-acetonitrile 1:1 (v/v)] containing 0.02 mg/ml of L-2-chlorophenylalanine as an internal standard. Then, after vortexing and mixing for 30 s, low-temperature ultrasonic extraction was performed for 30 min (5°C, 40 kHz). Next, the samples were incubated at −20°C for 30 min, followed by a 15-min centrifugation (13,000 g, 4°C). The resulting supernatants were collected and dried under a nitrogen stream. After drying, 120 μl of acetonitrile-water (1:1) was added for reconstitution, followed by vortex-mixing for 30 s; extraction was carried out by a 5-min low-temperature ultrasonication (5°C, 40 kHz). Finally, a 10-min centrifugation (13,000 g, 4°C) was performed, and supernatants were collected into injection vials for on-machine analysis. In addition, 20 µl supernatants from each sample were mixed as a quality control (QC) sample.

#### 2.5.2 Untargeted LC-MS Analysis

LC-MS was carried out in this study on a UHPLC-QExactiveHF-X system (Thermo Fisher Scientific). Chromatographic conditions were: column, ACQUITYUPLCHSST3 (100 mm × 2.1 mm, 1.8-µm i.d.) (Waters, Milford, United States); mobile phase A, 95% water and 5% acetonitrile (with 0.1% formic acid); mobile phase B, 47.5% acetonitrile, 47.5% isopropanol and 5% water (with 0.1% formic acid); injection volume, 2 μl; column temperature, 40°C. Mass spectrometry was performed in the positive and negative modes with electrospray ionization.

#### 2.5.3 Data Processing

Raw data were imported into ProgenesisQI (Waters Corporation, Milford, United States), a metabolomics software that performs baseline filtering, peak detection, integration, retention time correction, peak alignment and normalization. The resulting data matrix contains retention times, mass/charge ratios and peak intensities, among others. Then, the software was utilized for peak identification, matching MS and MS/MS data with the metabolic database. The MS mass error was < 10 ppm, and metabolite identification was performed according to the secondary mass spectrometry match scores. The Human Metabolome Database (HMDB, http://www.hmdb.ca/), METLIN database (https://metlin.scripps.edu/) and self-built databases were used for retrieval.

#### 2.5.4 Metabolomic Data Analysis

Principal component analysis (PCA) was carried out to detect outliers in rat urine samples and to distinguish the degree of similarity and difference in metabolic profiles of urine specimens among various groups. Next, orthogonal partial least squares discriminant analysis (OPLS-DA) was used for screening for metabolites that may help differentiate groups, parameters R2Y (cum) and Q2 (cum) were utilized to assess the quality of the OPLS-DA model, and metabolites with VIP > 1.2 and *p* < 0.05 were selected as potential molecular markers. Finally, metabolic pathways were determined based on differential metabolites, with *p* < 0.05 as a criterion. The analysis software in this study was the Majorbio Cloud Platform (www.majorbio.com). In addition, the potential molecular markers were imported into Cytoscape to generate a “Compound-Reaction-Enzyme-Gene” network.

### 2.6 Network Analysis

Firstly, the corresponding targets of paeoniflorin were determined using TCMSP (https://tcmsp-e.com/) and SwissTargetPrediction (http://www.swisstargetprediction.ch/). Depression-related targets were collected from the three databases GeneCards, OMIM and DRUGBANK, with “Depression” and “Depressive Disorder” as keywords, and the disease targets collected in the three disease databases were combined, and duplicate targets were deleted. The common target between the target of depression and the target of paeoniflorin was taken as a potential target of paeoniflorin in the treatment of depression. The potential targets were combined with related targets in the “compound-reaction-enzyme-gene” network as potential therapeutic targets of paeoniflorin. VENN diagrams were drawn using website (http://bioinformatics.psb.ugent.be/webtools/Venn/). Gene names were verified with Uniprot (https://www.uniprot.org/).

Secondly, potential therapeutic targets were submitted to STRING (https://string-db.org/) for protein-protein interaction (PPI) detection, and a PPI network was constructed by Cytoscape 3.7.1. Key targets were screened using the degree and betweenness of the plug-in model CytoHubba.

Finally, we used the OmicShare Tools (https://www.omicsshare.com/tools/home/report/goenrich.html) to perform biological pathway enrichment analysis to elucidate the function of each target and its function in signal transduction, including KEGG and GO analyses.

### 2.7 Molecular Docking

In this paper, AutoDock Vina v1.1.2 was used for molecular docking, and PyMol 2.5 was utilized to process all receptor proteins, including the removal of water molecules, salt ions and small molecules. Docking boxes were defined using PyMOL 2.5 so that all active pockets are wrapped. ADFRsuite 1.0 was used to convert all processed small molecules and receptor proteins to the PDBQT format necessary for AutoDock Vina 1.1.2 docking. The results were visualized and analyzed with PyMol 2.5.

### 2.8 Statistical Analysis

Data were analyzed with SPSS 20.0 and GraphPad Prism 8.0. All data were presented as mean ± standard deviation (SD). One-way analysis of variance (ANOVA) or nonparametric test was utilized for comparisons on the basis of data normality. *p* < 0.05 indicated statistical significance.

## 3 Results

### 3.1 Paeoniflorin Alleviates Depression-Like Behaviors in Chronic Unpredictable Mild Stress Rats

#### 3.1.1 Paeoniflorin Restores Body Weight in Chronic Unpredictable Mild Stress Rats

Body weight changes in rats of various groups are shown in Figure 1B. Before modeling, body weights were comparable in all groups (*p* > 0.05). From 1 to 3 weeks after modeling, in comparison with the NC group, body weights in the CUMS, FLU and PF groups were markedly decreased, with statistical differences (*p* < 0.01). After 1 week of drug treatment, in comparison with the CUMS group, body weights in the PF groups were increased, with statistically significant differences (*p* = 0.047); compared with the CUMS group, the FLU weight appeared to increase, but the difference was not statistically significant (*p* = 0.095). Following 2 weeks of drug administration, in comparison with the CUMS group, body weights in the PF and FLU groups were increased, with statistically significant differences (*p* = 0.012, *p* = 0.035). Following 3 weeks of drug administration, in comparison with the CUMS group, body weights in the FLU group were increased (*p =* 0.037), and body weights in the PF group were significantly increased (*p* = 0.006).

#### 3.1.2 Paeoniflorin Restores Total Distance in Chronic Unpredictable Mild Stress-Like Depressed Rats in an Open Field Test

The open field test was carried out before modeling, 3 weeks after modeling, and 6 weeks after modeling (Figure 1C). The total moving distances in various groups before modeling were roughly the same, with no statistically significant differences. There were differences in the total moving distances of rats at 3 weeks after modeling. In comparison with the NC group, total moving distances in the CUMS and PF group were reduced (*p* = 0.014, *p* = 0.037); in comparison with the NC group, total moving distances in the FLU groups were also markedly reduced (*p* = 0.003). After 6 weeks of modeling, the situation was reversed. In comparison with the CUMS group, total moving distances in the PF and PLU groups were markedly increased (*p* < 0.0001, *p* = 0.002).

#### 3.1.3 Paeoniflorin Responded to the Amount of Sugar Water Consumed by Chronic Unpredictable Mild Stress Rats

The sucrose preference test was performed prior to modeling, 3 weeks after modeling and 6 weeks after modeling (Figure 1D). Before the start of the experiment, sugar consumption rates of various groups of rats were roughly the same, with no statistical differences. After 3 weeks of modeling, there were differences in drinking sugar amounts in the rats. In comparison with the NC group, sugar consumption rates in the CUMS, FLU, and PF groups were markedly decreased, with statistical differences (*p* < 0.01). The situation was reversed following 3 weeks of continuous treatment with paeoniflorin and fluoxetine. In comparison with the CUMS group, sugar consumption rates in the PF and PLU groups were starkly increased, with statistically significant differences (*p* < 0.01).

### 3.2 Applying Metabolomics to Analyze Principal Components

**FIGURE 2:**
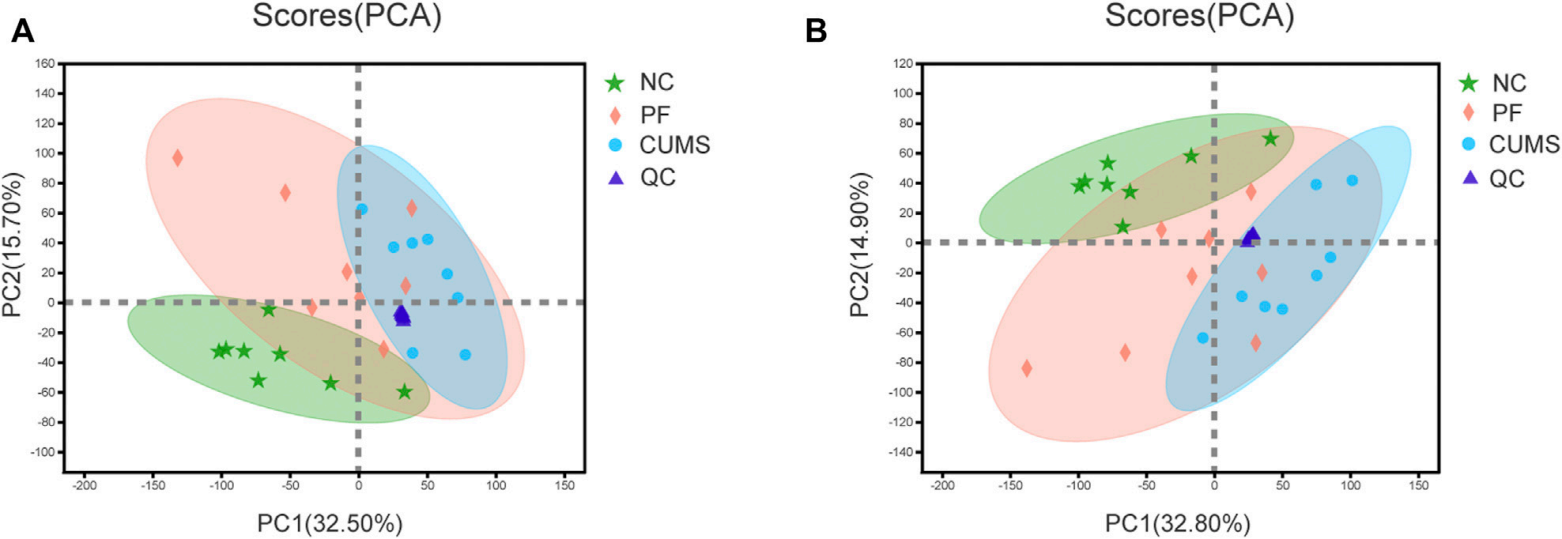
PCA score plot. Plot of PCA scores for the NC (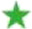), CUMS (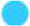) PF (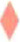) and QC (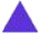) groups based on data acquired in positive and negative ion modes **(A,B)**.

The data of each group were assessed by unsupervised principal component analysis (PCA), and the NC, CUMS and PF groups were significantly separated. These trends indicated differences in metabolic profiles among groups, i.e., significant changes in urinary endogenous metabolites, as shown in Figure 2. There was a partial overlap between the NC and PF groups, which demonstrated after intervention with paeoniflorin in CUMS model rats, the metabolic state in the body tended to be close to that of the normal group. The PF group was distributed between the NC and CUMS groups, suggesting paeoniflorin has a marked regulatory effect on metabolic disorders triggered by CUMS-induced depression-like behavior. The QC samples showed good aggregation, and the results indicated that the entire analysis system had good stability and repeatability, meeting the requirements for metabolomics analysis.

**FIGURE 3:**
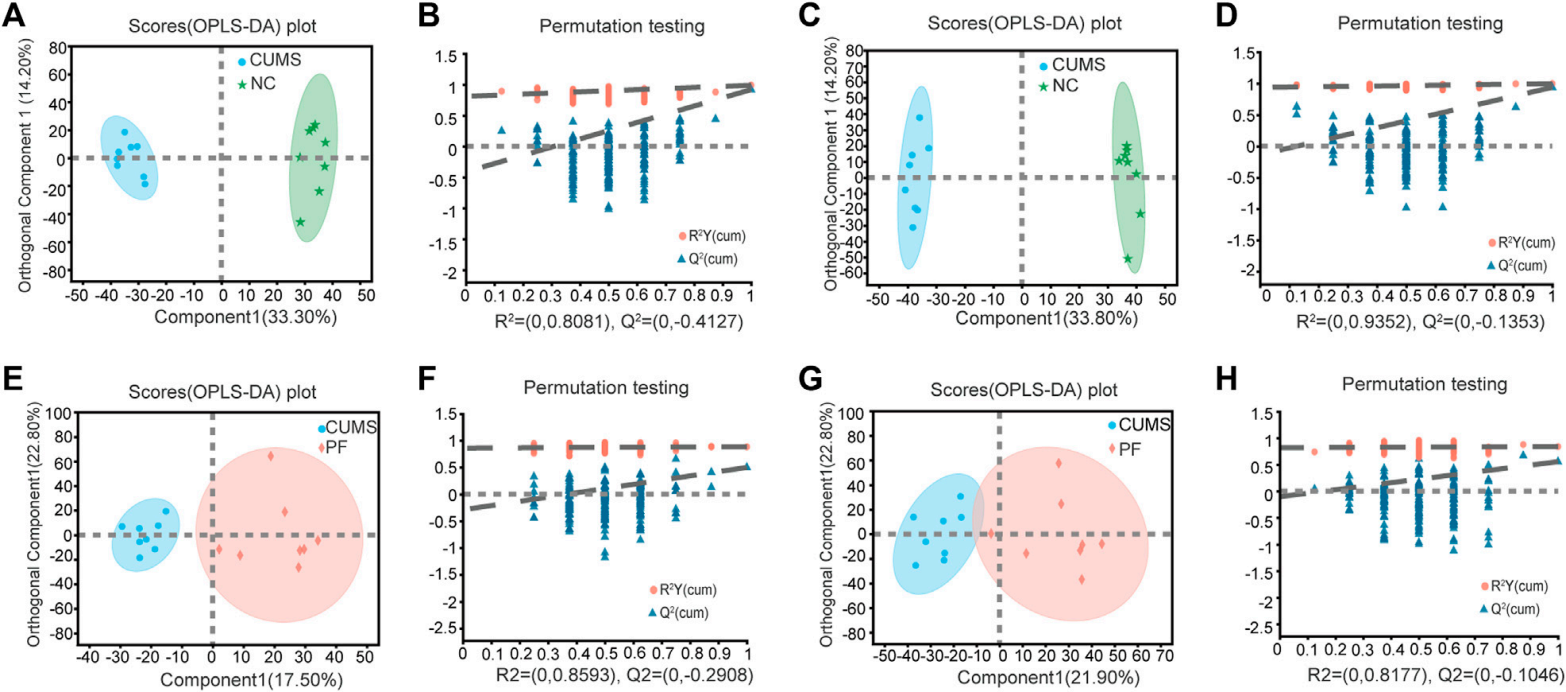
Multivariate data analysis of urine metabolites of rat samples. OPLS-DA score plot and permutation test of the NC and CUMS groups **(A,B)** in the positive ion mode and **(C,D)** in the negative ion mode. OPLS-DA score plot and permutation test of the CUMS and PF groups **(E,F)** in the positive ion mode and **(G,H)** in the negative ion mode.

OPLS-DA was used to further define potential biomarkers between group pairs. All loading plots were tested with 200 random permutations to ensure reliability and prevent overfitting. OPLS-DA of NC and CUMS groups showed that samples from the same group clustered together, and samples from different groups were well distinguished (Figures 3A,C). The random permutation test revealed R2X, R2Y and Q2 were 0.475, 0.988 and 0.917 in the positive mode, respectively (Figure 3B), and R2X, R2Y and Q2 were 0.603, 0.996 and 0.938 in the negative mode, respectively (Figure 3D). OPLS-DA of CUMS group and PF group showed that samples from the same group clustered together and samples from different groups distinguished well, as shown in Figures 3E,G. The random permutation test demonstrated R2X, R2Y and Q2 were 0.403, 0.883 and 0.498 in the positive mode, respectively (Figure 3F), and R2X, R2Y and Q2 were 0.453, 0.839 and 0.555 in the negative mode, respectively (Figure 3H). This shows that the model has good explanatory and predictive ability. Based on criteria of VIP > 1.2 and *p* < 0.05, differential metabolites were obtained as potential molecular markers, as shown in Table 2.

**TABLE 2:** Different endogenous metabolites in rat urine.

No.	Metabolites	Retention time (min)	Formula	CUMS/NC	PF/CUMS
1	Citric acid	1.3882	C6H8O7	↓**	↑^##^
2	12-oxo-5E,8E,10Z-dodecatrienoic acid	4.9219	C12H16O3	↑**	↓^##^
3	(+/-)-Hexanoylcarnitine	4.3347	C13H25NO4	↑**	↓^##^
4	Blumenol C glucoside	4.7034	C19H32O7	↑**	↓^#^
5	(1R,4R)-Dihydrocarvone	5.9385	C10H16O	↑**	↓^#^
6	S-Nitrosoglutathione	2.8026	C10H16N4O7S	↑**	↓^#^
7	(R)-(-)-Mellein	1.7491	C10H10O3	↓**	↑^##^
8	1-(Malonylamino)cyclopropanecarboxylic acid	1.8034	C7H9NO5	↓**	↑^##^
9	Biopterin	1.8238	C9H11N5O3	↑**	↓^##^
10	{[1-(2-hydroxyphenyl)-3-oxopropan-2-yl]oxy}sulfonic acid	3.0543	C9H10O6S	↓**	↑^##^
11	6-{4-[(1E)-3-[(3-carboxy-5,6-dihydroxycyclohex-3-en-1-yl)oxy]-3-oxoprop-1-en-1-yl]-2-hydroxyphenoxy}-3,4,5-trihydroxyoxane-2-carboxylic acid	3.1494	C22H24O14	↓**	↑^##^
12	3-carboxy-4-methyl-5-pentyl-2-furanpropanoic acid	3.3253	C14H20O5	↑**	↓^##^
13	Alanyl-Proline	4.2321	C8H14N2O3	↑**	↓^##^
14	Tranylcypromine glucuronide	4.2526	C15H19NO6	↑**	↓^##^
15	(Z)-3-Oxo-2-(2-pentenyl)-1-cyclopenteneacetic acid	4.4713	C12H16O3	↑**	↓^#^
16	3-Hydroxytetradecanedioic acid	5.0311	C14H26O5	↑**	↓^##^
17	O-Desmethylvenlafaxine glucuronide	5.8158	C20H29NO8	↑**	↓^#^
18	10-Hydroxymyoporone	5.9385	C15H22O4	↑**	↓^#^
19	13Z-Docosenamide	6.5628	C22H43NO	↓**	↑^#^
20	5-(hydroxymethyl)-2-Furancarboxylic acid	9.9531	C6H6O4	↓**	↑^##^
21	N-Acetyltyramine	5.6997	C10H13NO2	↑**	↓^##^
22	Tsangane L 3-glucoside	5.5905	C19H34O7	↑**	↓^##^
23	4-Isopentylphenol	5.5768	C11H16O	↑**	↓^#^
24	Trigoforin	5.4881	C12H12O2	↑**	↓^##^
25	2-{4-[(1E)-1,2-diphenylbut-1-en-1-yl]phenoxy}ethan-1-ol	5.1541	C24H24O2	↓**	↓^#^
26	5-NITRO-2-PHENYLPROPYLAMINOBENZOIC ACID [NPPB]	4.7374	C16H16N2O4	↑**	↓^##^
27	Dihydromaleimide beta-D-glucoside	4.6628	C10H15NO7	↓**	↑^#^
28	3,8-Dihydroxy-6-methoxy-7 (11)-eremophilen-12,8-olide	4.6559	C16H24O5	↑**	↓^##^
29	Cis-2,3-Dihydroxy-2,3-dihydro-p-cumate	4.2526	C10H14O4	↓**	↑^##^
30	Erysonine	4.1433	C17H19NO3	↑**	↓^##^
31	4-hydroxy-5-(4-hydroxy-3-methoxyphenyl)pentanoic acid	3.8706	C12H16O5	↑**	↓^#^
32	2,6-Dioxo-6-phenylhexanoate	3.7752	C12H12O4	↑**	↓^##^
33	Thioacetate	3.693	C2H4OS	↑**	↓^#^
34	(5R)-5-Hydroxyhexanoic acid	3.2167	C6H12O3	↑**	↓^#^
35	Veranisatin A	3.1494	C16H22O8	↓**	↑^##^
36	5-Hydroxy-2-(5-methyl-1-oxo-4-hexenyl)benzofuran	2.3819	C15H16O3	↑**	↓^#^
37	2-Methoxyhydroquinone	2.2798	C7H8O3	↓**	↑^#^
38	5′-Deoxyadenosine	1.9395	C10H13N5O3	↑*	↓^##^
39	N-(2-Hydroxyethyl)iminodiacetic acid	0.6368	C6H11NO5	↓**	↑^#^
40	Quinone	2.0213	C6H4O2	↓**	↑^#^
41	Stachyose	0.8169	C24H42O21	↓**	↑^##^
42	Thiamine monophosphate	1.9084	C12H17N4O4PS	↓**	↑^##^
43	Gluconolactone	2.7931	C6H10O6	↓**	↑^##^
44	4-Hydroxy-5-(phenyl)-valeric acid-O-sulphate	4.0312	C11H14O6S	↑**	↓^#^
45	Beta-D-Glucopyranosyl-11-hydroxyjasmonic acid	4.2413	C18H28O9	↑**	↓^##^
46	Valproic acid glucuronide	5.2494	C14H24O8	↑**	↓^##^
47	9,15-dioxo-11R-hydroxy-2,3,4,5-tetranor-prostan-1,20-dioic acid	5.5638	C16H24O7	↓**	↓^##^
48	{[1-(4-methoxyphenyl)-4-methylpent-1-en-3-yl]oxy}sulfonic acid	5.487	C13H18O5S	↑**	↓^##^
49	Xi-3-Hydroxy-5-phenylpentanoic acid O-beta-D-Glucopyranoside	5.3754	C17H24O8	↑**	↓^##^
50	Alpha-CEHC glucuronide	5.0472	C22H30O10	↑**	↓^#^
51	6-(4-ethyl-2-methoxyphenoxy)-3,4,5-trihydroxyoxane-2-carboxylic acid	4.9778	C15H20O8	↑**	↓^#^
52	1,4-Ipomeadiol	3.9826	C9H14O3	↑**	↓^#^
53	{4-[(E)-2-{3,5-dihydroxy-4-[(1E)-3-methylbuta-1,3-dien-1-yl]phenyl}ethenyl]phenyl}oxidanesulfonic acid	3.4771	C19H18O6S	↓**	↑^#^
54	5-Hydroxyindoleacetic acid	3.3727	C10H9NO3	↓**	↑^##^
55	Trans-Chlorogenic acid	2.8821	C16H18O9	↓*	↓^##^
56	3,4,5-trihydroxy-6-[(2-hydroxy-2-methylpropanoyl)oxy]oxane-2-carboxylic acid	1.6639	C10H16O9	↑**	↓^#^

↑ represents up-regulation, ↓ represents down-regulation. **p < 0.01 and *p < 0.05, CUMS group versus NC group. ^##^ *p* < 0.01 and ^#^ *p* < 0.05, PF group versus CUMS group.

**FIGURE 4:**
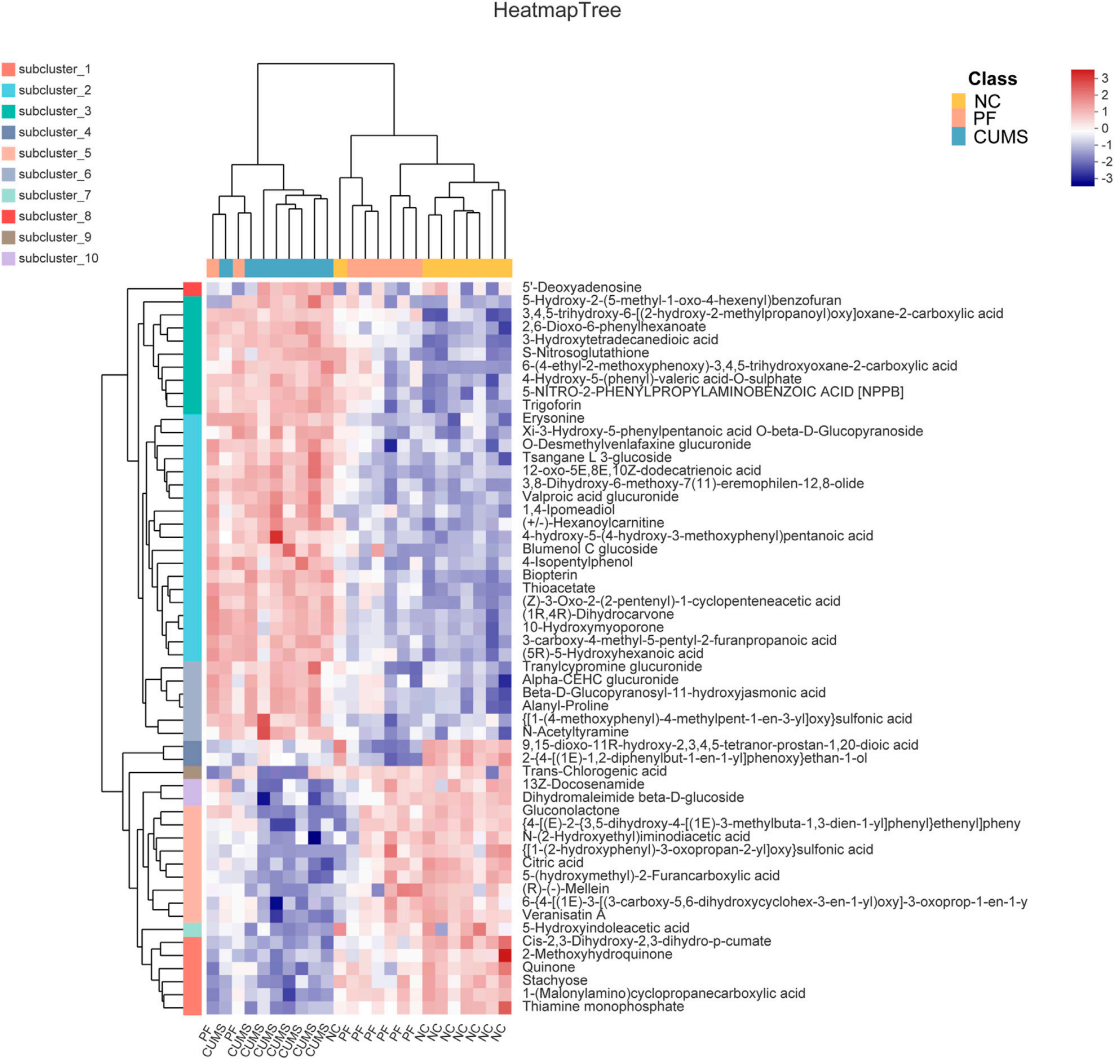
Comparison of differentially expressed metabolite levels between different groups. Each row corresponds to data for a specific metabolite, and each column represents the NC, CUMS, or PF group. Colors indicate the relative expression levels of metabolites in this group of samples.

Among these biomarkers, except 2-{4-[(1E)-1,2-diphenylbut-1-en-1-yl]phenoxy}ethan-1-ol, 9,15-dioxo-11R-hydroxy-2,3,4,5-tetranor-prostan-1,20-dioic acid and Trans-Chlorogenic acid, the levels of 53 biomarkers in CUMS rats were partially reversed by paeoniflorin. Figure 4 shows a heatmap of the relative intensities of biomarkers and the separation between different groups, especially paeoniflorin with a modulating effect on CUMS rats. We found significant changes in the CUMS group compared to the NC group, with elevated levels of 35 metabolites, such as (+/-)-Hexanoylcarnitine, Blumenol C glucoside, and Biopterin. There were decreased levels of 18 metabolites such as Citric acid, Thiamine monophosphate, Gluconolactone, 5-Hydroxyindoleacetic acid. Paeoniflorin significantly restored the levels of Citric acid, Thiamine monophosphate, Gluconolactone, 5-Hydroxyindoleacetic acid and other metabolites in CUMS rats after intervention.

### 3.3 Analysis of Paeoniflorin-Related Metabolites and Metabolic Pathways

**FIGURE 5:**
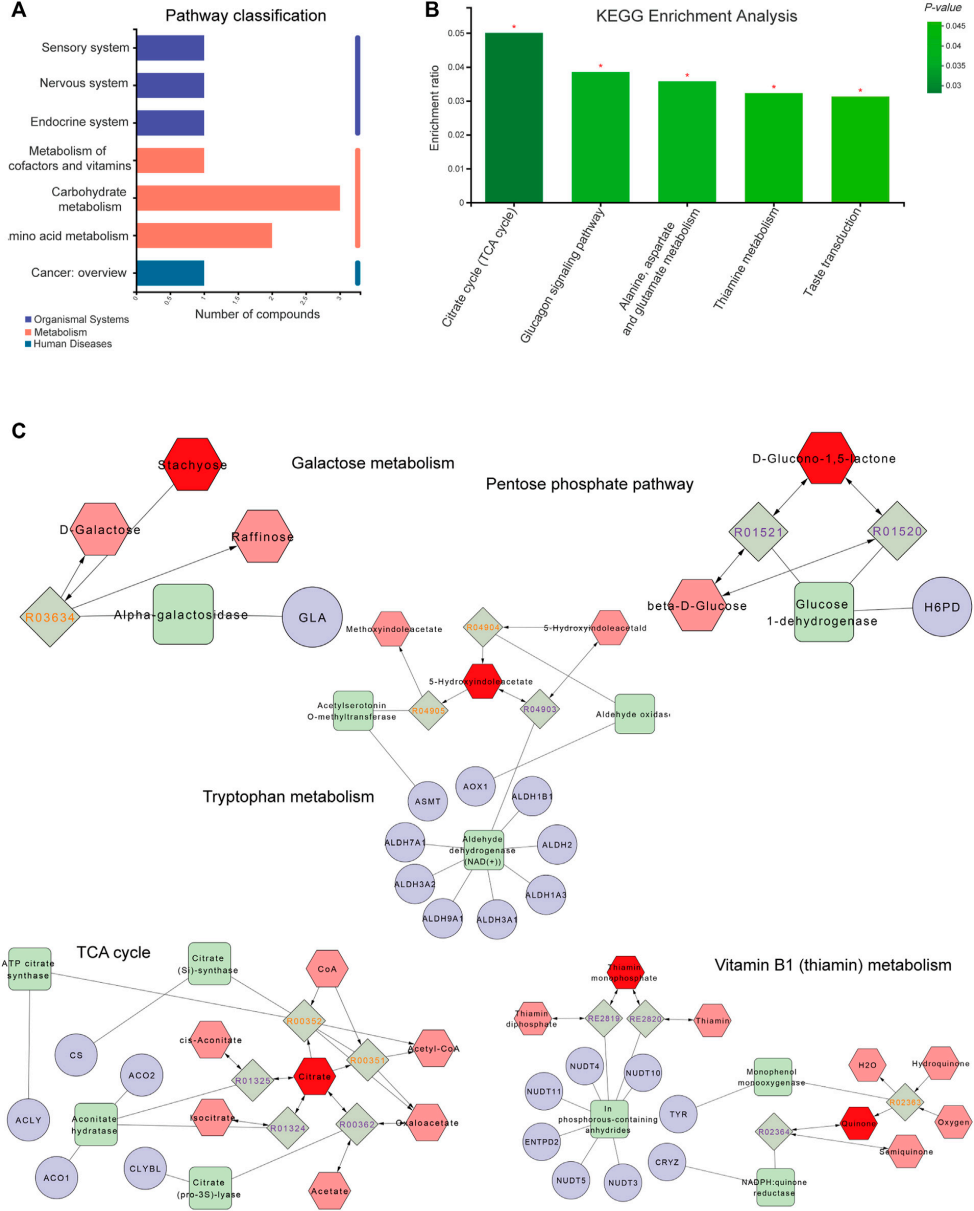
Metabolite pathway analysis and networks. **(A)** The ordinate and abscissa represent the secondary classification of a KEGG metabolic pathway and the amount of related metabolites, respectively. **(B)** The abscissa and ordinate represent the pathway’s name and enrichment rate, respectively. **(C)** Compound-reaction-enzyme-gene network. Red hexagons, gray diamonds, green rectangles and purple circles are active compounds, reactions, enzymes and genes, respectively. *p < 0.05, **p < 0.01 and ***p < 0.001.

As shown in Figures 5A,B, metabolic pathways including differential metabolites were assessed through the Majorbio Cloud Platform. The KEGG pathways including differential metabolites were counted, among which the carbohydrate metabolism pathway had the most differential metabolites. KEGG analysis was carried out for pathways comprising differential metabolites, and *p* < 0.05 was used as the screening basis to obtain five important metabolic pathways, including the citrate cycle (TCA cycle), glucagon signaling pathway, thiamine metabolism, taste transduction and alanine, aspartate and glutamate metabolism. The relevant metabolites involved were citric acid, thiamine monophosphate, gluconolactone and 5-hydroxyindoleacetic acid.

### 3.4 Metabolomics Combined With Network Analysis to Predict the Possible Mechanisms Involved in Paeoniflorin

To fully understand the mechanism by which paeoniflorin alleviates depression, differential metabolites were imported into the MetScape plugin of Cytoscape to generate a “Compound-Reaction-Enzyme-Gene” network, and an interaction network combining metabolomics and network pharmacology was built (Figure 5C). The results demonstrated related targets included *NUDT4*, *NUDT5*, *NUDT3*, *CRYZ*, *CS*, *NUDT10*, *CLYBL*, *ALDH2*, *ALDH3A1*, *ALDH1B1*, *ALDH1A3*, *ALDH9A1*, *ALDH3A2*, *GLA*, *AOX1*, *ASMT*, *ACLY*, *ACO1*, *ACO2*, *ALDH7A1*, *NUDT11*, *TYR*, *ENTPD2* and *H6PD*. Relevant metabolites were thiamin monophosphate, D-glucono-1,5-lactone, stachyose, citrate and 5-hydroxyindoleacetate. The relevant metabolic pathways were the TCA cycle, Vitamin B1 (thiamin) metabolism, tryptophan metabolism, pentose phosphate pathway and galactose metabolism. Combined with KEGG analysis results of differential metabolites, the TCA cycle was the key metabolic pathway of paeoniflorin in the treatment of depression, and citric acid, thiamine monophosphate, gluconolactone, 5-hydroxyindoleacetic acid and stachyose were the key metabolites of paeoniflorin in the treatment of depression.

### 3.5 Chemical Composition Network Analysis

#### 3.5.1 Potential Targets of Paeoniflorin in Depression Therapy

**FIGURE 6:**
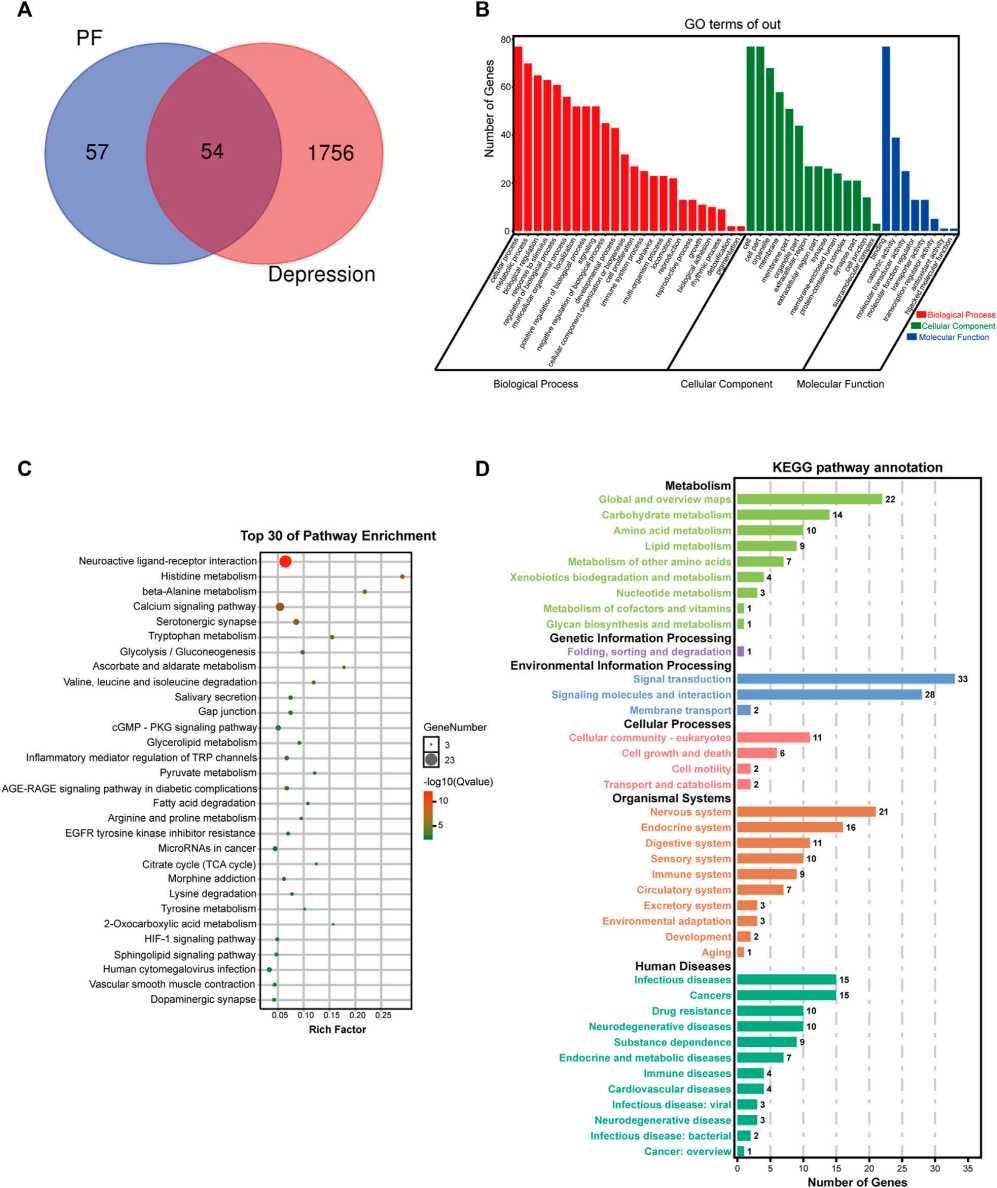
Potential target network analysis. **(A)** Venn diagram of potential targets. **(B)** GO analysis of potential targets. **(C)** Top 30 KEGG pathways of potential targets. **(D)** KEGG pathway annotation.

First, paeoniflorin’s targets were searched in the TCMSP database and SwissTarget website, and 111 targets were retrieved. Totally 1810 depression disease targets were obtained by searching the GeneCards, DRUGBANK, OMIM and TTD databases. The targets of paeoniflorin and the disease targets of depression were combined, and common targets were considered the possible targets of paeoniflorin in depression treatment, 54 in total. A VENN diagram was drawn (Figure 6A). The potential targets were merged with related targets in the “Compound-Reaction-Enzyme-Gene” network, a total of 78 potential targets were used for subsequent analysis.

#### 3.5.2 Pathway Prediction of Paeoniflorin by KEGG and GO

To elucidate the biological characteristics of potential targets, GO enrichment analysis was performed with the OmicShare Tools. Totally 46 GO terms were detected, including 24 biological process (BP), 8 molecular function (MF) and 14 cellular component (CC) terms (Figure 6B). BP terms involved multiple genes were significantly enriched in cellular process, metabolic process, biological regulation, response to stimulus, multicellular organismal process, localization, signaling, developmental process, cellular component organization or biogenesis, cell proliferation, immune system process, locomotion, etc. In addition, CC and MF terms associated with paeoniflorin’s antidepressant effect were cell, organelle, membrane, extracellular region, synapse, membrane-enclosed lumen, protein-containing complex, binding, catalytic activity, molecular transducer activity, molecular function regulator, antioxidant activity, etc.

To further explore these potential targets, KEGG analysis was carried out to illustrate the functional activities involved in paeoniflorin’s therapeutic effect on depression. Figure 6C shows the top 30 signaling pathways obtained by KEGG analysis, as well as the classification statistics of signaling pathways enriched by KEGG analysis (Figure 6D). The results of KEGG enrichment analysis showed the main pathways associated with paeoniflorin’s antidepressant effect included neuroactive ligand-receptor interaction, histidine metabolism, calcium signaling pathway, beta-alanine metabolism, serotonergic synapse, tryptophan metabolism, glycolysis/gluconeogenesis, ascorbate and aldarate metabolism, cGMP-PKG signaling pathway, inflammatory mediator regulation of TRP channels, citrate cycle (TCA cycle), HIF-1 signaling pathway and sphingolipid signaling pathway. The B-class classification statistics of various pathways showed that paeoniflorin mainly exerts antidepressant effects through activities such as the nervous system, carbohydrate metabolism, amino acid metabolism and signal transduction.

#### 3.5.3 Protein-Protein Interaction Network and Major Antidepressant Targets of Paeoniflorin

**FIGURE 7:**
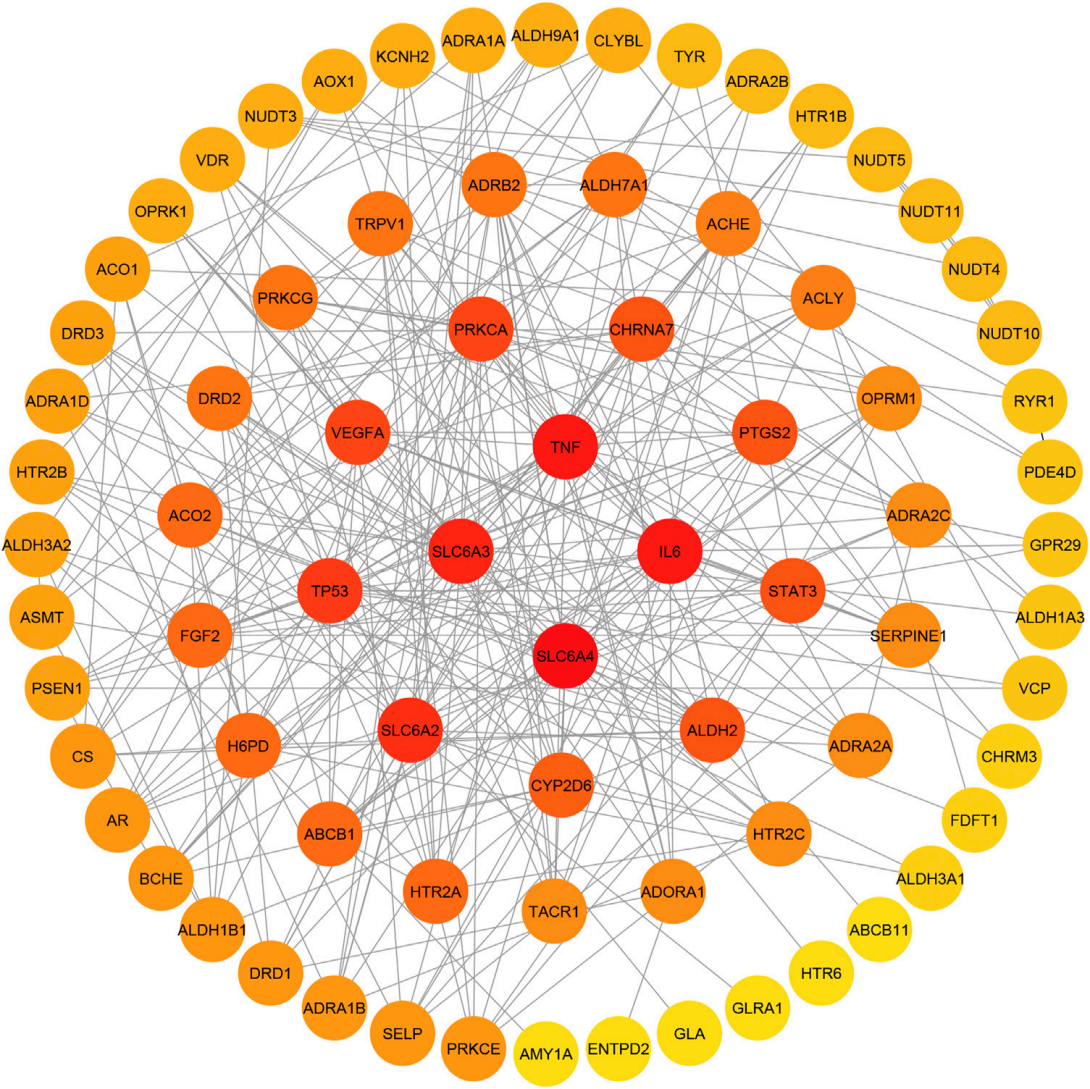
Potential target interaction network (PPI). The color of the circle changes from red to yellow to represent the change of the degree value of the target point from large to small; the four target points in the center have the largest degree values.

The protein-protein interaction relationships of potential targets were obtained based on the STRING database. This PPI network consisting of 78 nodes and 293 edges. The information was imported into CytoScape 3.7.1 to visualize the PPI network relationship, and the CytoHubba function was used to calculate degree values. The results showed that the four targets with the largest degree values were *SLC6A4*, *TNF*, *IL6* and *SLC6A3*. Therefore, these four targets were considered key targets of paeoniflorin in the treatment of depression, as shown in Figure 7.

### 3.6 Components-Targets Docking Analysis

**FIGURE 8:**
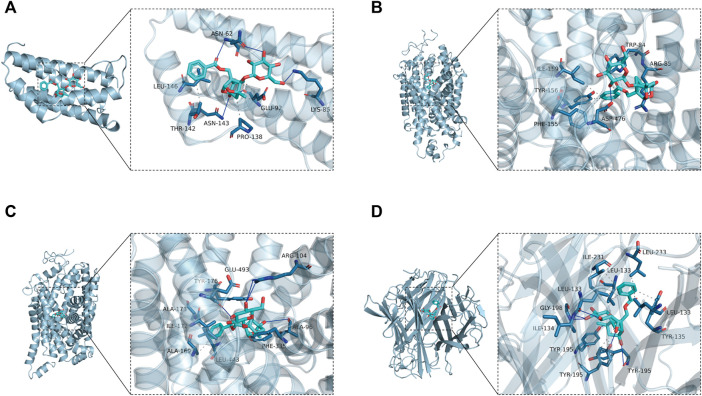
Molecular docking-predicted binding mode. **(A)** Molecule docking of PF binding to *IL6*. **(B)** Molecule docking of PF binding to *SLC6A3*. **(C)** Molecule docking of PF binding to *SLC6A4*. **(D)** Molecule docking of PF binding to *TNF*. Left panel, overall view; right panel, partial view; cyan stick, small molecule; light blue cartoon, protein; blue line, hydrogen bond; gray dotted line, hydrophobic interaction.

According to the generated PPI network, the four key targets were used for molecular docking. The docking binding of paeoniflorin to each target is shown in Table 3. Molecular docking showed that paeoniflorin had good binding activity to key targets. In particular, the binding energy of paeoniflorin to *SLC6A4* was −10.2 kcal/mol, suggesting that the combination is closely bound and has high activity potential. The binding mode of paeoniflorin to key targets is shown in Figure 8.

**TABLE 3:** Molecular docking score (kcal/mol).

Target name	Ligand name	Docking score
*IL6*	Paeoniflorin	−7.3
*SLC6A3*	Paeoniflorin	−7.6
*SLC6A4*	Paeoniflorin	−10.2
*TNF*	Paeoniflorin	−8.6

As shown in Figure 8A, paeoniflorin binds to the middle of the *IL6* helix, and forms hydrogen bonds with ASN-62, ASN-143 and LYS-85 on the protein. In addition, it also forms hydrophobic interactions with GLU-92, PRO-138, THR-142 and LEU-146. Hydrogen bonding and hydrophobic interactions are the main forces that maintain the binding of small molecules and proteins. As shown in Figure 8B, paeoniflorin can form hydrogen bonds with ASP-476 and ARG-85 on the protein. In addition, it can also form hydrophobic interactions with ILE-159, TYR-156, PHE-155 and TRP-84 on the protein to further strengthen the binding between the molecule and the protein. As shown in Figure 8C, similar to paeoniflorin/*SLC6A3*, paeoniflorin also binds inside the helix of the protein, and the binding is maintained mainly by hydrogen bonds and hydrophobic interactions. For example, small molecules form hydrogen bonds with TYR-176, ARG-104 and ALA-96 in proteins. It also forms hydrophobic interactions with ALA-173, ILE-172, ALA-169, LEU-443, PEH-335 and TYR-176. As shown in Figure 8D, paeoniflorin binds to the inner active channel formed by *TNF* trimers, and can form hydrogen bonds with LEU-133, ILE-134 and TYR-195 in the channel. It can also form hydrophobic interaction with LEU-133, ILE-231, LEU-233, LEU-133 and TYR-195 in proteins.

## 4 Discussion

Depression is the most common mental disorder in clinical. Depression is largely underdiagnosed and rarely treated due to stigma, lack of effective treatments and insufficient mental health resources (Smith 2014). In recent years, the prevalence of depression has increased year by year in different age groups, making it one of the most important global health problems (Park et al., 2019). Current treatments for depression include antidepressants and somatic therapy (Voineskos et al., 2020). Among them, antidepressants are the most commonly used treatments for patients with depression. However, it is known that only 20%–40% of depressed patients respond to low doses of these drugs, and the risk of adverse effects increases with higher doses (de Oliveira et al., 2022). Studies have found that herbal medicine has a strong antidepressant effect with few adverse reactions (Wang et al., 2017; Chi et al., 2019). In our previous experiments, we confirmed that paeoniflorin is one of the main components of Xiaoyaosan, a classic antidepressant herbal medicine (Ding et al., 2020).

In this study, we explored the targets, efficacy and underlying mechanisms of paeoniflorin in the treatment of depression through metabolomics and network analysis. To verify the antidepressant effect of paeoniflorin, CUMS combined with Orphan was established to induce depression in rats. As previously reported by others, CUMS is currently a well-recognized animal model of depression, and the animal model established in combination with orphanage can stably simulate the core symptom of human depression, namely anhedonia (Willner et al., 1987; Hill et al., 2012; Hu et al., 2017; Zheng et al., 2021). In the present study, CUMS led to a decrease in the sucrose preference rate in SPT and a decrease in total travel distance in OFT, which could be reversed by paeoniflorin. Depression is often related to loss of appetite, fatigue and anhedonia, showing that CUMS-induced changes in these parameters are similar to clinical symptoms of depression (Goldstein Ferber et al., 2021). These results demonstrate that the antidepressant-like effects of paeoniflorin are associated with chronic stress-induced decreased motor activity and anhedonia in rats.

In an LC-MS-based untargeted urine metabolomic analysis, we identified 56 metabolic biomarkers. CUMS rats had reduced levels of 18 metabolites, such as Citric acid, Quinone, Thiamine monophosphate, and 5-Hydroxyindoleacetic acid, which were consistent with findings on metabolite levels in other depression (Zhang et al., 2013; Gao et al., 2014; Ding et al., 2015; Sun et al., 2022). After paeoniflorin intervention, 18 metabolites decreased in CUMS rats were upregulated, and they exerted antidepressant effects mainly by regulating citrate cycle (TCA cycle), glucagon signaling pathway, thiamine metabolism, taste transduction and amino acid metabolism.

Multiple studies have reported an association between the TCA cycle and depression. Some researchers believe that the TCA cycle is specific and blocked in depression (Mishra et al., 2020; Ting et al., 2020; Tian et al., 2022). Mitochondrial dysfunction or changes in brain energy metabolism may be involved in the pathogenesis of depression (Bansal and Kuhad 2016; Kolar et al., 2021). Citric acid is a key intermediate in the TCA cycle and part of the energy metabolism pathway. In the present study, we found that the levels of citric acid were decreased in depressed rats compared with controls. In contrast, PF significantly upregulated abnormal citric acid levels in the urine of depressed rats. It suggested that paeoniflorin improved the biomarkers of TCA cycle in CUMS-induced depression in rats.

Studies have shown that the pathogenesis of depression is related to the dysfunction of amino acid metabolism (Nagasawa et al., 2012). Glutamate is an important excitatory neurotransmitter that plays a crucial role in regulating neuroplasticity, learning and memory (Deutschenbaur et al., 2016). Chronic stress reduces the structure and function of glutamatergic neurons, resulting in reduced volume of cortical and limbic structures associated with depression (Duman et al., 2019). Glucagon signaling pathway mainly assists glucagon to play a role in raising blood sugar, which can promote the catabolism of glucose. A genome-wide study of suicidal behaviors in depression found that the Glucagon signaling pathway was most abundant in major depression disorder (Zhao et al., 2020). Bendix et al. (2017) found that lower glucagon levels in plasma and cerebrospinal fluid were associated with suicidal behavior (Bendix et al., 2017). Thiamine is essential for normal brain function, and its deficiency leads to metabolic disturbances, specific lesions, oxidative damage, and reduced hippocampal neurogenesis (Vignisse et al., 2017). Zhang et al. (2013) reported a negative correlation between thiamine nutritional status and depressive symptoms in Chinese elderly (Zhang et al., 2013). A transcriptomic analysis found that the taste transduction pathway is downregulated in female unipolar depression, and the authors suggest that the decreased taste perception in depressed patients is attributable to decreased expression levels of taste transduction pathway genes (Dmitrzak-Weglarz et al., 2021).

Network analysis showed that SLC6A4, TNF, IL6, and SLC6A3 were the key targets of paeoniflorin for its antidepressant effect. Molecular docking showed the interaction mode between paeoniflorin and the target protein, and both paeoniflorin and the target protein had good binding activity. The docking results initially revealed the material basis of paeoniflorin in the treatment of depression. *SLC6A4* has been shown to be associated with psychiatric disorders, can be used to explain the association between major depressive disorder and suicidal tendencies, and is the most promising therapeutic target for future depression research (Park et al., 2019; Hande et al., 2021). *TNF* is an important cytokine contributing to the inflammatory hypothesis of depression, has an important function in the pathophysiology of depression, and represents a potential marker of depression (Liu et al., 2020). In a meta-analysis, it was shown that depression can be predicted by measuring *IL6* (Colasanto et al., 2020). *SLC6A3* can affect depressive symptoms and is a novel candidate biomarker gene for suicidal behavior (Rafikova et al., 2021). Therefore, the reliability of our findings can be fully demonstrated based on the above literature evidence.

## 5 Conclusion

In the present work, paeoniflorin’s antidepressant effect on CUMS-associated depression was systematically investigated by combining LC-MS-based metabolomics with network pharmacology analysis. Totally 56 biomarkers were identified, with significant changes involved in citrate cycle (TCA cycle), glucagon signaling pathway, thiamine metabolism, taste transduction, vitamin B1 (thiamin) metabolism, tryptophan metabolism, pentose phosphate pathway, galactose metabolism and alanine, aspartate and glutamate metabolism. Moreover, citric acid, thiamine monophosphate, gluconolactone, 5-hydroxyindoleacetic acid and stachyose were selected as key markers, and *SLC6A4*, *TNF*, *IL6*, and *SLC6A3* were identified as key targets, which may be related to the antidepressant effect of paeoniflorin. Molecular docking validated the key targets showed good affinities with PF.

To the best of our knowledge, this study provides the first demonstration that PF regulates multiple biological pathways and metabolic changes to attenuate depression-like symptoms by influencing the key target proteins. These findings contribute to a deeper understanding of the mechanism of paeoniflorin in the treatment of CUMS rats. However, we performed multivariate statistical analysis only by untargeted metabolomics. Also, we did not screen for metabolic biomarkers in CUMS rat brain tissue. Therefore, future studies will focus on validating the results by targeted metabolomics and screening metabolic biomarkers in the brain tissue of paeoniflorin-treated CUMS rats. Furthermore, in-depth validation studies are warranted to better understand the regulatory mechanism of paeoniflorin, which is expected to help develop paeoniflorin as a potential complementary drug for the treatment of depression.

## Data Availability

The original contributions presented in the study are included in the article/supplementary material, further inquiries can be directed to the corresponding authors.
